# Supplementary material for: Impact of Different Layer Housing Systems on Eggshell Cuticle Quality and Salmonella Adherence in Table Eggs
Source: Foods. 2021 Oct 23;10(11):2559. doi: 10.3390/foods10112559 (PMC8625084; doi:10.3390/foods10112559)
Supplement: Supplementary file 1 [file foods-10-02559-s001.zip › foods-1413390-supplementary.pdf]

Supplementary Figures and Tables

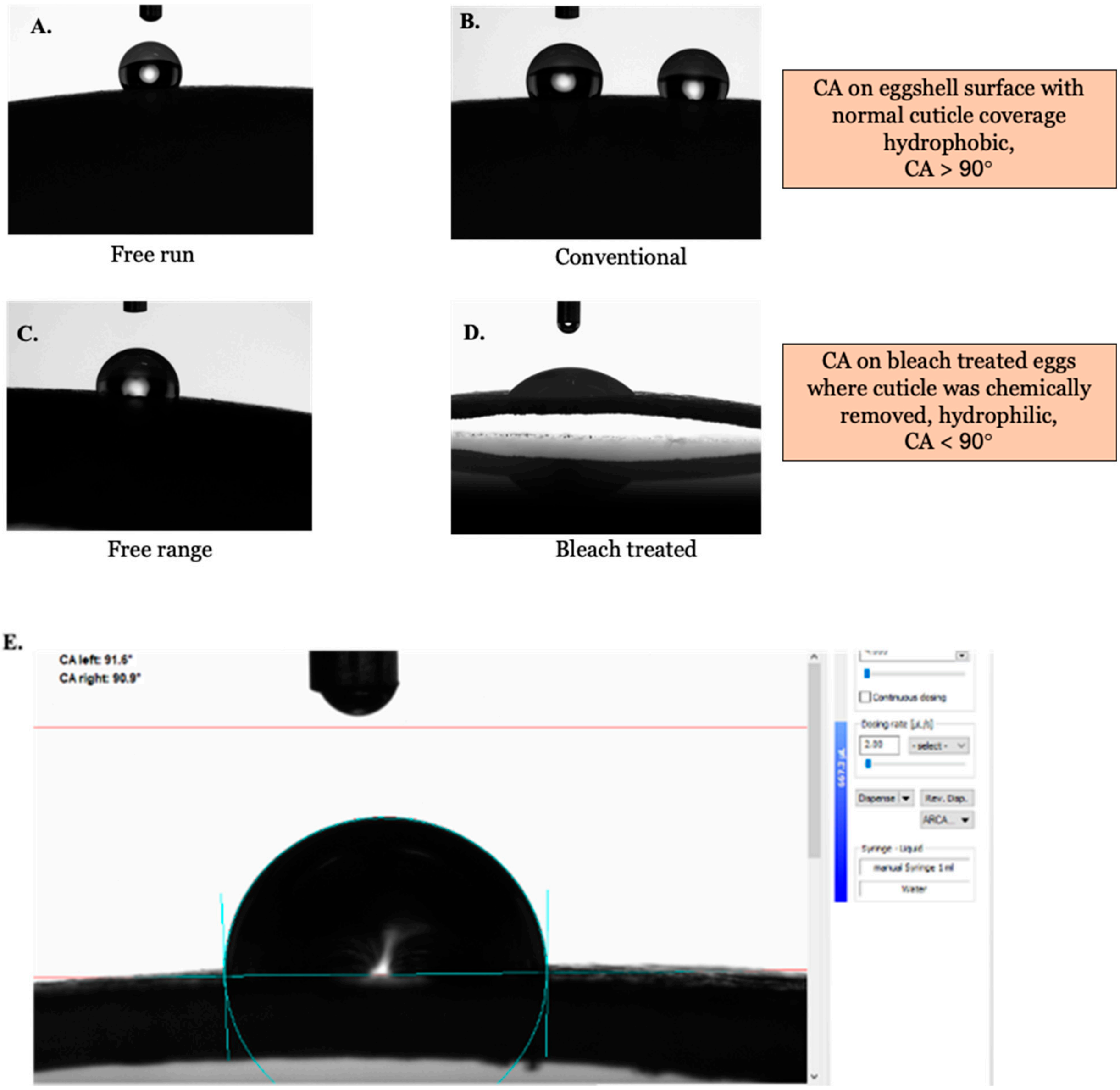

Figure S1. Contact angle measurement using droplets of deionized water on eggshell surface of eggs from different housing systems including A. Free run, B. Conventional, C. Free range and D. No cuticle, and E. Measurement of contact angle of a droplet (on eggshell from free range eggs) using the SCA20 software (Dataphysics). Each CA value is the mean of left and right measurements.

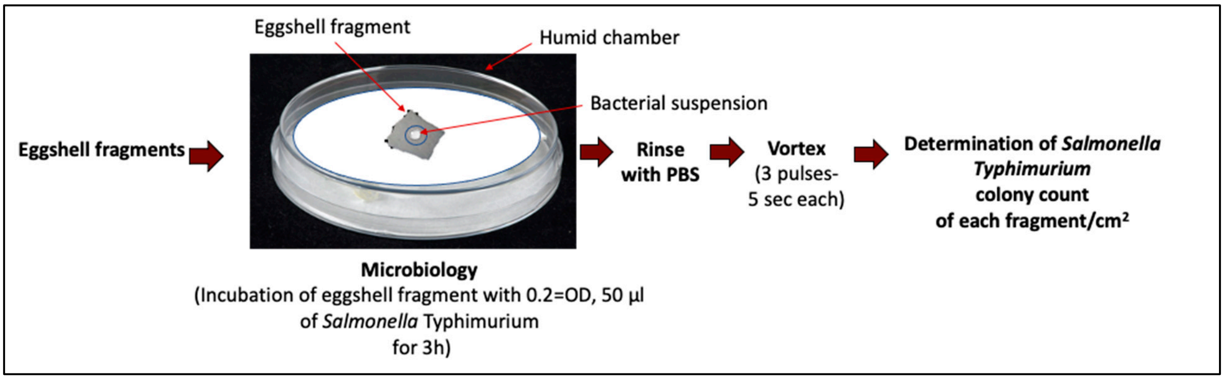

Figure S2. Schematic representation of bacterial cell attachment assay to determine *Salmonella* cell counts on the outer surface of eggshell from eggs from different housing system.

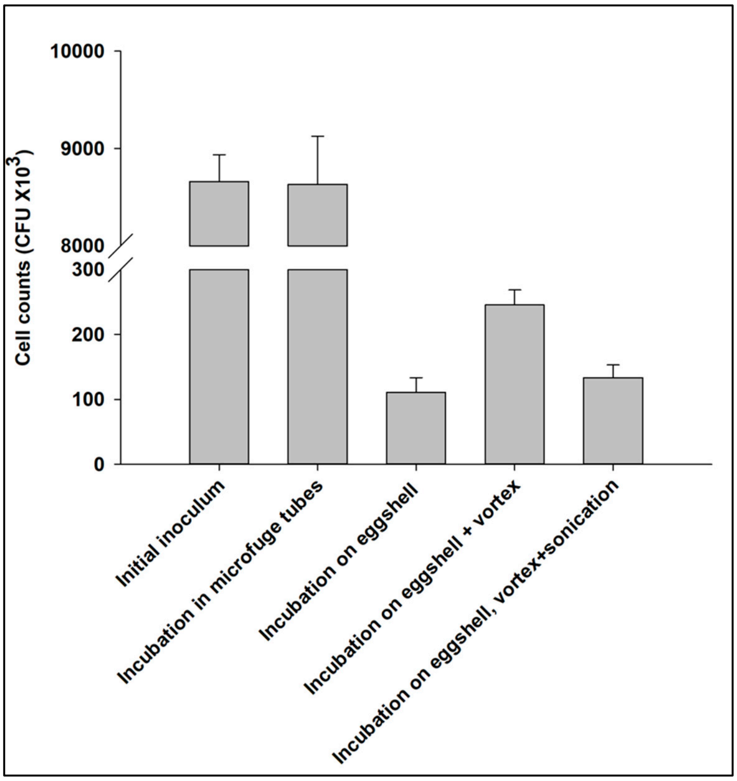

Figure S3. Preliminary data showing a comparison of bacterial cell counts in initial inoculum vs incubated on eggshell surface. Vortexing was more effective in dislodging *Salmonella* cells from the outer surface of no cuticle (bleach treated) eggshells as compared to vortex + sonication.

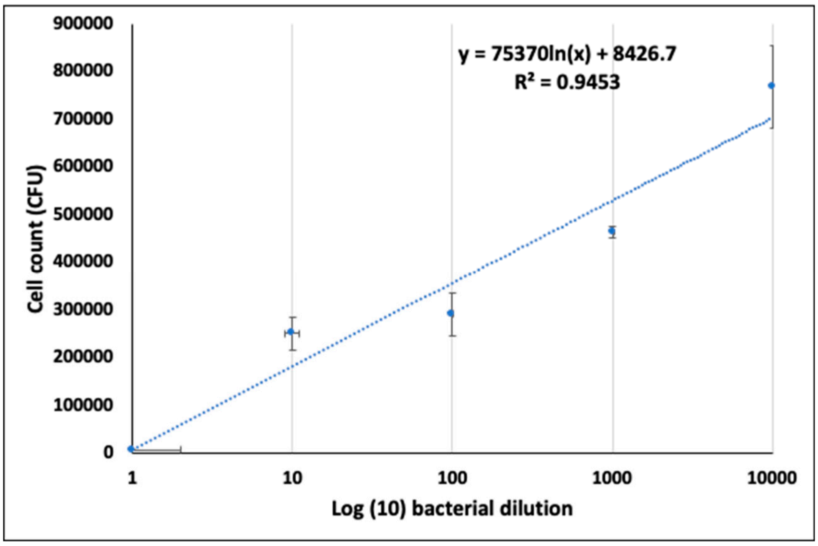

Figure S4. Bacterial cell attachment assay. Scatter plot on a logarithmic scale showing a correlation between adhering *Salmonella* cell counts and serially diluted bacterial dose applied to the eggshell surface.

TableS1. Elemental analysis of cuticle pore plugs of eggs from different housing system analyzed by energy-dispersive x-ray spectroscopy (EDS).

Elements[1]	Housing systems (Normalized weight %)					
	**Conventional**	**Enriched**	**Free run**	**Free range**	**Pooled StDev**	***P-value***
**C**	22.91	24.37	25.16	20.95	4.55	0.417
**O**	44.78	39.03	41.43	40.01	6.96	0.518
**Na**	0.31	0.33	0.02	0.38	0.29	0.183
**Mg**	1.27	1.37	0.93	1.45	0.46	0.245
**P**	4.53	4.65	3.31	4.40	1.49	0.415
**S**	0.15	0.18	0.13	0.36	0.24	0.405
**Cl**	0.57	3.07	0.30	0.96	2.71	0.305
**K**	0.38	0.27	0.33	1.11	0.53	0.05
**Ca**	25.07	26.66	28.35	30.36	10.13	0.824

[1]Values represent mean from 18 replicates (n=3 x 6) from each housing system.

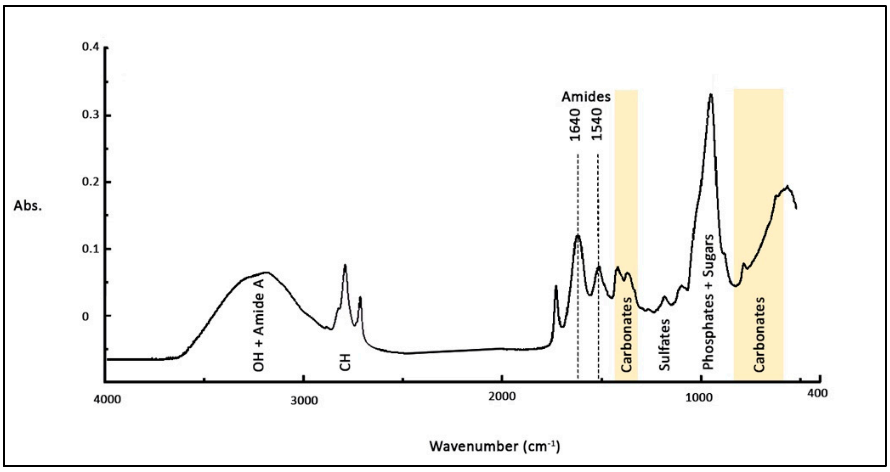

Figure S5. Attenuated total reflection-Fourier transform infrared spectra in the 4000 to 400 cm^{-1} range of the eggshell surface.

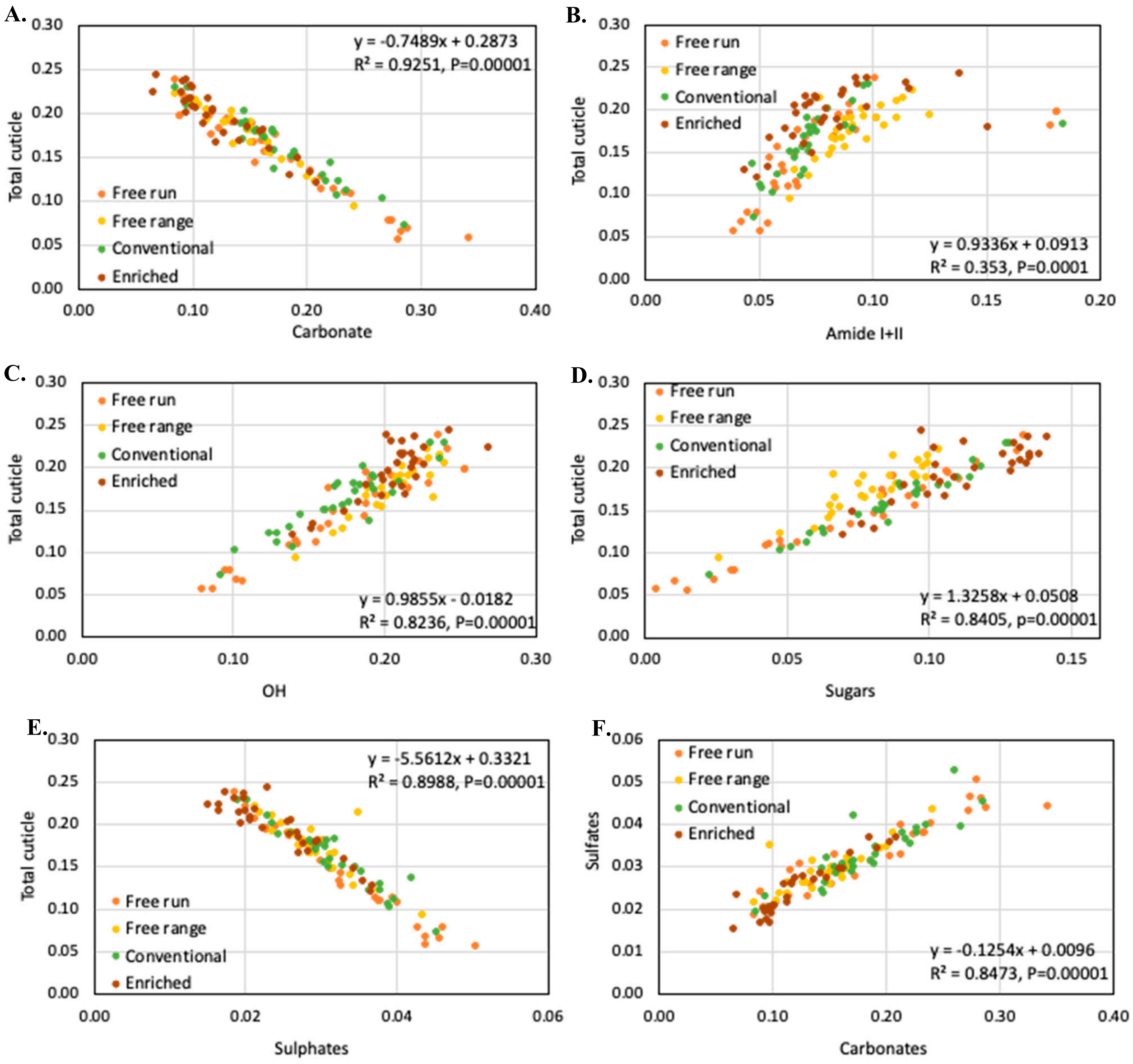

Figure S6. The correlation between cuticle chemical components A. Total cuticle vs carbonates, B. Total cuticle vs amides, C. Total cuticle vs OH, D. Total cuticle vs Sugar, E. Total cuticle vs sulfates, F. Sulfates vs carbonates, of eggshell cuticle established using linear regression ($Y_i=\beta_0+\beta_1X_i$) in a curve fitting model using excel.

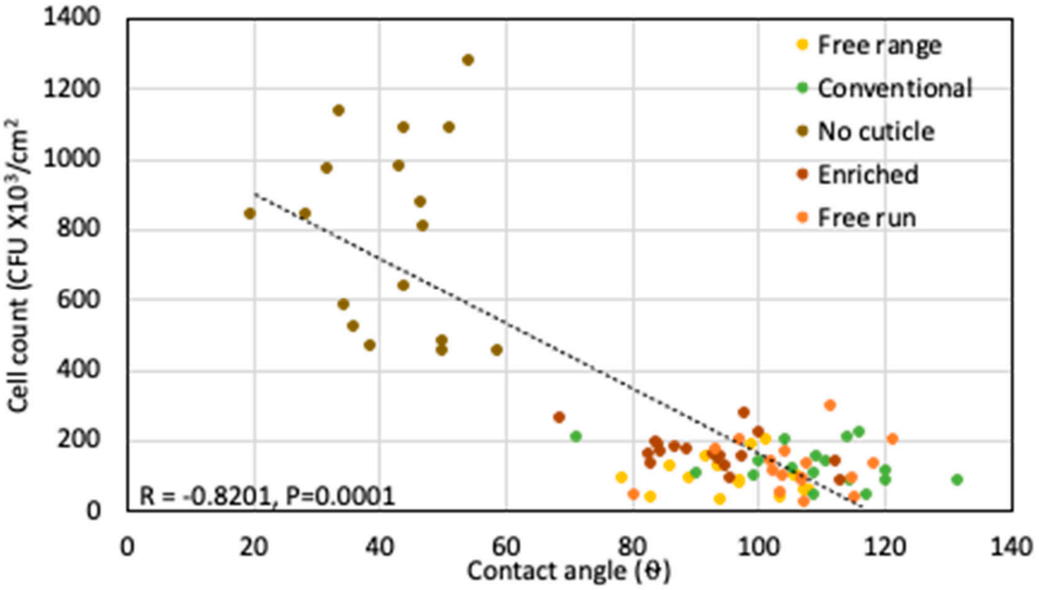

Figure S7. Pearson correlation analysis demonstrates a negative correlation between contact angle and *Salmonella* adherence on the eggshell surface. The correlations were established using linear regression ($Y_i=\beta_0+\beta_1X_i$) in a curve fitting model using excel.
